# Immunosuppressive Treatment for Nephrotic Idiopathic Membranous Nephropathy: A Meta-Analysis Based on Chinese Adults

**DOI:** 10.1371/journal.pone.0044330

**Published:** 2012-09-05

**Authors:** Guoqiang Xie, Jing Xu, Chaoyang Ye, Dongping Chen, Chenggang Xu, Li Yang, Yiyi Ma, Xiaohong Hu, Lin Li, Lijun Sun, Xuezhi Zhao, Zhiguo Mao, Changlin Mei

**Affiliations:** Kidney Institute of CPLA, Division of Nephrology, Changzheng Hospital, Second Military Medical University, Shanghai, China; Pennington Biomedical Research Center, United States of America

## Abstract

**Background:**

Idiopathic membranous nephropathy (IMN) is the most common pathological type for nephrotic syndrome in adults in western countries and China. The benefits and harms of immunosuppressive treatment in IMN remain controversial.

**Objectives:**

To assess the efficacy and safety of different immunosuppressive agents in the treatment of nephrotic syndrome caused by IMN.

**Methods:**

PubMed, EMBASE, Cochrane Library and *wanfang*, *weipu, qinghuatongfang*, were searched for relevant studies published before December 2011. Reference lists of nephrology textbooks, review articles were checked. A meta-analysis of randomized controlled trials (RCTs) meeting the criteria was performed using Review Manager.

**Main Results:**

17 studies were included, involving 696 patients. Calcineurin inhibitors had a better effect when compared to alkylating agents, on complete remission (RR 1.61, 95% CI 1.13, to 2.30 P = 0.008), partial or complete remission (effective) (CR/PR, RR 1.29, 95% CI 1.09 to 1.52 P = 0.003), and fewer side effects. Among calcineurin inhibitors, tacrolimus (TAC) was shown statistical significance in inducing more remissions. When compared to cyclophosphamide (CTX), leflunomide (LET) showed no beneficial effect, mycophenolate mofetil (MMF) showed significant beneficial on effectiveness (CR/PR, RR: 1.41, 95% CI 1.16 to 1.72 P = 0.0006) but not significant on complete remission (CR, RR: 1.38, 95% CI 0.89 to 2.13 P = 0.15).

**Conclusions:**

This analysis based on Chinese adults and short duration RCTs suggested calcineurin inhibitors, especially TAC, were more effective in proteinuria reduction in IMN with acceptable side effects. Long duration RCTs were needed to confirm the long-term effects of those agents in nephrotic IMN.

## Introduction

Idiopathic membranous nephropathy (IMN) is the most common cause of nephrotic syndrome for adults in western counties, as well as in China. Although 30% patients showed spontaneous complete or partial remission of nephrotic syndrome [1], 30–40% of patients progress toward end-stage renal disease (ESRD) within 5–15 years [2].

A meta-analysis [3] included 18 worldwide RCTS have been made to assess the effects and safety of immunosuppressive treatment of nephrotic idiopathic membranous nephropathy (IMN) in 2004, glucocorticoids improved proteinuria but did not induce remission. Combined corticosteroids with cytotoxic agents showed effectiveness to nephrotic IMN patients in many trials [4]–[7] and was considered a standard treatment.

Immunosuppressive treatment has been widely used in the treatment of IMN worldwide. However, there are still big controversies over the efficacy and safety of different immunosuppressive agents treatments in IMN, especially for those relatively new agents like, tacrolimus (TAC) and leflunomide (LET). So a meta-analysis comparing the efficacy and safety of different immunosuppressive agents in the treatment of Chinese adults with nephrotic IMN makes sense. China is the country with largest population of the world. To exclude the interferences caused by the ethnic variety, this meta-analysis was made on Chinese adults base.

## Methods

### Information Sources and Search Strategy

We tried to include all the RCTs that assess the efficacy and tolerability associated with the comparison of different immunosuppressive agents for the treatment of Chinese adults with nephrotic IMN. PubMed (up to December 2011), EMBASE (1980 to December 2011), and Cochrane Library (Issue12, 2011) and the databases in Chinese including *wanfang*, *weipu, qinghuatongfang* (up to December 2011) were searched, and reference lists of nephrology textbooks, review articles were checked.

### Inclusion Criteria

1. Prospective RCTs compared different immunosuppressive agents.
2. The selected patients were Chinese adults suffering from IMN, aged 16 years or older, with nephrotic syndrome.
3. The diagnosis of IMN was made by renal needle biopsy.

### Exclusion Criteria

1. Study design without randomization, own control or compared with different usage of the same agent.
2. Secondary types of membranous nephropathy or not Chinese patients.
3. Trials including the use of traditional Chinese medicine were excluded,for its unknown additional effects on immunosuppressive agents and uncertain dose of active components. We also excluded studies where it was impossible to identify how many patients had nephrotic syndrome, after checking the baseline evaluations and contacting with the authors.

### Study Selection

Two reviewers(G. Xie and J. Xu) independently assessed the eligibility of each article to be included in this meta-analysis, and this work was checked by another author (Z. Mao).

### Data Collection Process and Data Items

Data were extracted from each identified trial by two researchers (G. Xie and J. Xu) with a predesigned review form (Microsoft Office Excel 2007) independently, and any disagreement was resolved by discussion. Authors of the original studies were consulted through emails for suggestions if any problem occurred.

The following data were included: the authors of each study, the year of publication, the design of the trial, the duration of the study, the sample size, the age and gender of the patients, the interventions (mainly immunosuppressive agents, dose and usage), the baseline proteinuria/serum creatinine/serum albumin values, the final proteinuria/serum creatinine/serum albumin values, and the therapeutic remission of participants (complete remission, partial remission). In addition, we retrieval the side effects including elevated liver enzymes, renal toxicity, infections, digestive symptoms, leukocytopenia, and other recorded.

### Risk of Bias

The quality of included studies were evaluated by two authors (C Ye and D Chen) independently based on the standard criteria (randomization, blinding, and loss to follow-up)using the scoring system developed by Jadad [8]. The quality scoring system was as follows: (1) Was the study described as randomized? (2 = Properly with detailed description of randomization, 1 = randomized but detail not reported); (2) Was the blind method used? (2 = Double-blind, 1 = single-blind, 0 = open-label); (3) Were dropout and follow-up reported? (1 = Numbers and reasons reported, 0 = not reported). The publication bias was assessed by examining the funnel plot. A sensitivity analysis was performed by omitting low quality studies and investigating the influence on the overall meta-analysis estimate.

### Data Analysis and Statistical Methods

Statistical analyses were performed with Review Managerver 5.0.20 (Cochrane Collaboration, Oxford, UK). We assessed the heterogeneity of the trial results by calculating a chi-square test of heterogeneity and the I^2^measure of inconsistency. Dichotomous data were summarized as risk ratio (RR) and 95% confidence intervals (CIs), continuous ones (final proteinuria) as weighted mean difference (WMD) and 95% CIs as well.

**Figure 1:**
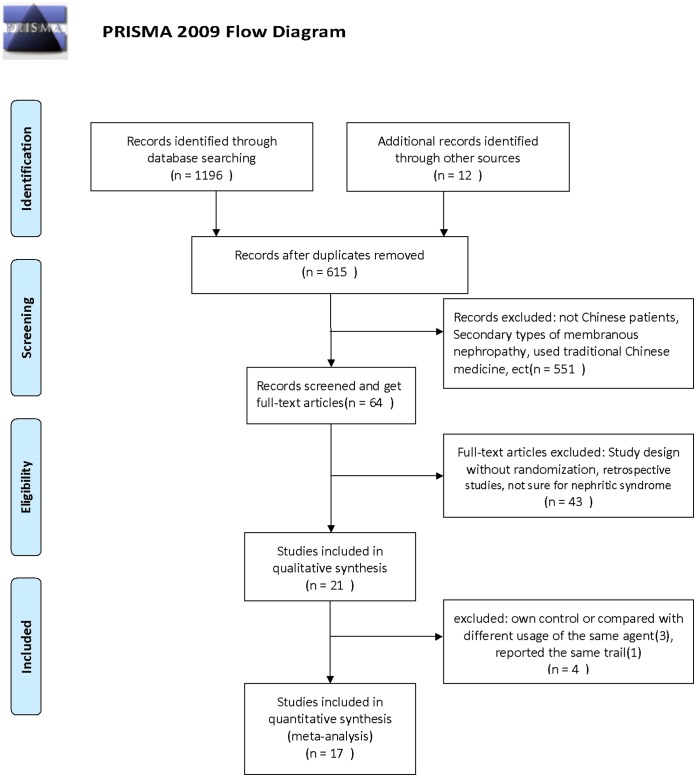
PRISMA Flowchart.

The Flowchart of this meta-analysis was shown in Figure 1.

## Results

### Study Characteristics

All included trials were prospective RCTs, 3 [9]–[11] were published in English and 14 were published in Chinese. The included studies involved 696 patients. Only one study [11] used blindness and it is the only one published as conference abstract without full text. In 15 of 17 studies, cyclophosphamide(CTX) was involved in the comparison. 6 studies compared MMF with alkylating agents, 5 of them with CTX, the other one with chlorambucil. 7 studies compared calcineurin inhibitors with alkylating agents (only CTX). 3 studies compared leflunomide (LET) with CTX. 1 study compared LET with TAC. Characteristics of the included trials are shown in Table 1.

**Table 1:** Characteristics of the included trials.

Trials	Number	Length	Mean age(year)	Gender male/female	Baseline proreinuria(g/day)	Initial steroids dose	Quality grade
CyA versus CTX							
Li GF 2011 [14]	76	12 months	45.2/44.8	49/27	5.4±2.3/5.0±2.1	PDN0.5 mg/kg/d	2
Wu QX 2011 [16]	40	12 months	36.2	29/11	6.2±3.5/5.9±4.1	aPDN0.8 mg/kg/d	2
LET versus CTX							
Li GF 2011 [24]	80	6 months	48.3/47.6	63/17	3.59±1.18/3.72±1.23	PDN0.5 mg/kg/d	2
Zhou W 2009 [22]	30	12 months	42.8/41.6	15/15	7.84±3.73/7.78±3.67	Prednisolone 0.8–1.0 mg/kg/d	3
Zhu KY 2009 [23]MMF versus CTX	40	>6 months	51	24/16	6.15±2.36/6.17±2.53	aPDN30 mg/d	2
Zhang W 2011 [20]	60	12 months	43.6/43.6	38/22	7.55±3.66/7.48±3.63	PDN0.5/1.0 mg/kg/d	3
Zhou W 2009 [21]	40	12 months	43.8/42.6	17/23	7.93±3.82/7.62±3.55	Prednisolone 0.8–1.0 mg/kg/d	3
Li MX 2004 [18]	40	12 months	45.5	29/11	5.01±1.78/5.15±1.87	PDN1.0 mg/kg/d	2
An WW 2009 [17]	32	12 months	53.6	20/12	8.4±2.2/NC	Prednisolone60 mg/d	2
Ren Y 2011 [19]	52	12 months	46.6/41.1	36/16	NC	PDN0.8–1.0 mg/kg/d	1
TAC versus CTX							
Bai GZ 2011 [12]	32	9 months	48.2	21/11	NC	PDN15–60 mg/d	1
Xu J 2010 [11]	24	24 months	55.0/54.6	15/9	NC	NC	>3
Chen M 2010 [10]	73	12 months	47.2/48.6	41/32	7.11±3.93/7.28±3.91	PDN1 mg/kg/d	3
Chen WZ 2009 [13]	17	9 months	NC	NC	4.0±0.7/3.9±1.6	PDN15–60 mg/d	2
Liu JP 2009 [15]	20	6 months	51.3	13/7	NC	PDN1 mg/kg/d	2
MMF versus chlorambucil							
Chan TM 2007 [9]	20	15 months	49.5	13/7	4.9(3.4–6.9)/5.8(4.1–8.1)median (range)/median(range)	Prednisolone 0.8/mPDN1g×3 days thenPrednisolone 0.4 mg/kg/d	3
TAC versus LET							
Sun GD 2008 [25]	20	6 months	49.5	14/6	9.87±2.45/8.96±1.79	PDN30 mg/d	2

Abbreviations: PDN, prednisone; aPDN, prednisone acetate; NC, not clear.

### Effects of Interventions

#### Calcineurin inhibitors versus alkylating agents

Seven trials [10]–[16] involving 282patients compared calcineurin inhibitors with alkylating agents, 5 [10]–[13], [15] for comparing TAC with CTX, 2 [14], [16] for comparing CyA with CTX. Calcineurin inhibitors showed statistically significant higher rate on inducing remission, on complete remission (CR, RR: 1.61, 95% CI 1.13 to 2.30, P = 0.008) figure 2.1, on complete/partial remission (CR/PR, RR: 1.29, 95% CI 1.09 to 1.52, P = 0.003) figure 2.2.

**Figure 2:**
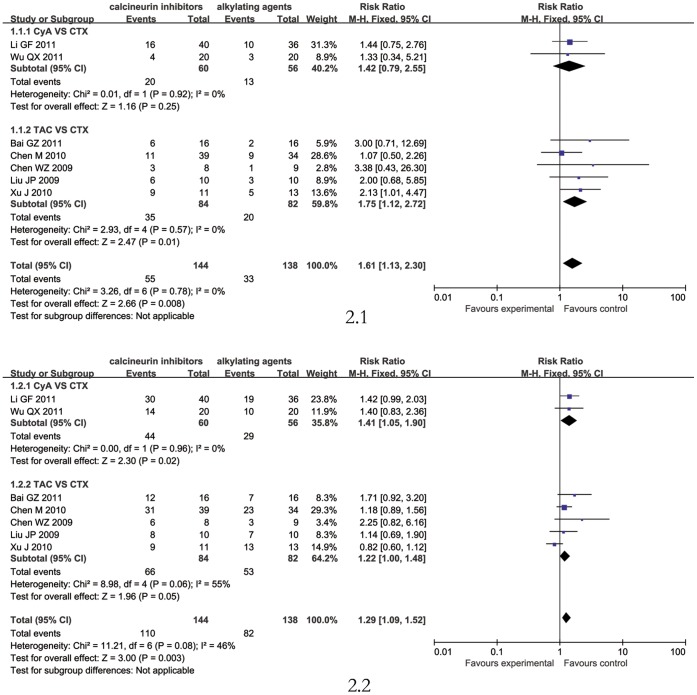
The complete remission rate (CR; **figure 2**.1) and the complete/partial remission rate (CR/PR; **figure 2**.2) comparison between calcineurin inhibitors and alkylating agents.

### Comparison of Two Agents

#### MMF versus CTX

5 studies [17]–[21] involving 224 patients compared MMF with CTX. MMF was given at 1.0–2.0 g/d orally for the first period (3 to 6 months), and then gradually tapered. Immunosuppressive treatment lasted for 12 months. MMF showed significant benefit on effectiveness (CR/PR, RR: 1.41, 95% CI 1.16 to 1.72, P = 0.0006) figure 3.2 but no significant on complete remission (CR, RR: 1.38, 95% CI 0.89 to 2.13, P = 0.15) [figure 3.1].

**Figure 3:**
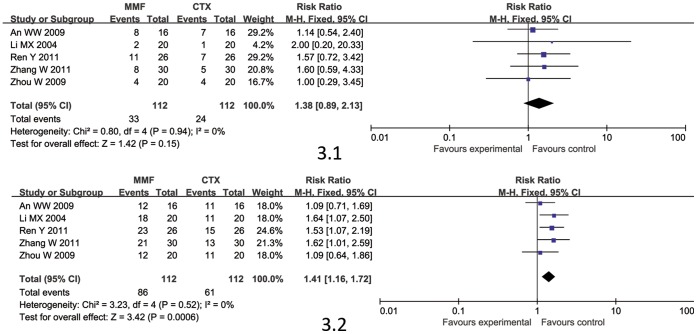
The complete remission rate (CR; **figure 3**.1) and complete/partial remission rate (CR/PR; **figure 3**.2) comparison between MMF and CTX.

#### TAC versus CTX

5 studies [10]–[13], [15] involving 166 patients compared TAC with CTX. TAC was given at 0.1 mg/kg/d initially and adjusted to a blood trough concentration level at 5 to 10 ng/mL for the first period (mostly 6 months), then reduced. TAC induced more remission than CTX (CR, RR 1.75, 95% CI 1.12 to 2.72, P = 0.01; CR/PR, RR 1.22, 95% CI 1.00 to 1.48, P = 0.01) (figure 2), with lower final proteinuria (WMD 1.12, 95% CI 0.53 to 1.71).

#### LET versus CTX

**Figure 4:**
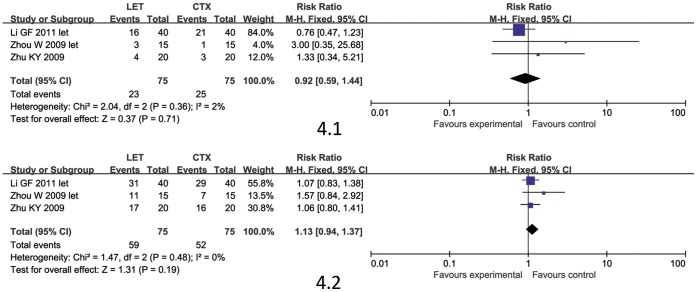
The complete remission rate (CR; **figure 4**.1) and the complete/partial remission rate (CR/PR; **figure 4**.2) comparison between LET and CTX.

3 studies [22]–[24] involving 150 patients compared LET with CTX. LET was given orally 50 mg/d for 3 days, followed by 20–30 mg/d for 3 months, and then tapered. LET showed no benefit in inducing remissions compared to cyclophosphamide. (CR, RR 0.92, 95% CI 0.59 to 1.44, P = 0.71; CR/PR, RR 1.13,95% CI 0.94 to 1.37, P = 0.19) figure 4.

#### CyA versus CTX

Only 2 studies [14], [16] involving 116 patients compared CyA with CTX. CyA was given at 3–5 mg/kg/d initially and adjusted to a blood trough concentration level at 100 to 200 µg/L during the induction period (all 3 months),then tapered the doses. CyA showed better responsiveness (CR/PR, RR: 1.41, 95% CI 1.05 to 1.90, P = 0.002) figure 2.2 but no significant on complete remission (CR, RR: 1.42, 95% CI 0.79 to 2.55, P = 0.25) figure 2.1.

#### TAC versus LET

Only 1 study [25] involving 20 patients compared TAC with LET. TAC was given at 0.1 mg/kg/d initially and adjusted to a blood trough concentration level at 5 to 10 ng/mL for 6 months; LET was given orally at 50 mg/d for 3 days, then 50 mg/d for 6 months, 1 hour before breakfast. TAC showed borderline advantage on complete remission (CR, RR: 2.50, 95% CI 0.63 to 10.00, P = 0.20), and on complete/partial remission (CR/PR, RR: 1.80, 95% CI 0.94 to 3.46, P = 0.08).

### Side Effects

As side effects in a single comparison was not easy to make a statistical analysis, the major side effects of each agents were showed as following. 325 patients were given cyclophosphamide in total, and adverse events in 309 patients were reported: 42(13.6%) with dysfunction of liver, 37(12.0%) with leukocytopenia, 28(9.1%) with digestive symptoms. Hypertrichosis was the most frequent side effect of CyA (9/60, 15%). Elevated blood glucose happened in 18/78(23.1%) patients treated with TAC, 3 of which developed diabetes mellitus. 8/78(10.3%) patients treated with TAC got elevated blood pressure, and were treated with increased anti-hypertension drugs.

Eight among 112 (7.1%) patients given MMF got digestive symptoms. 6/75(8.0%) patients given LET got elevated liver enzymes, anther 8% got digestive symptoms.

There was no obvious nephrotoxicity directly related to immunosuppressive agents. 3 patients reported transient elevation of Scr in the comparison of “TAC versus CTX”, 2 for CTX, 1 for TAC, and none of them progressed to renal failure. Sun GD et al [25] reported increased serum creatinine concentration in 5 patients, 2 for TAC and 3 for LET. The very high proteinuria in this study (mean 9.87 g/24 h in TAC group, 8.96 g/24 h in LET group) should be taken into account.

### Sensitivity Analysis

**Figure 5:**
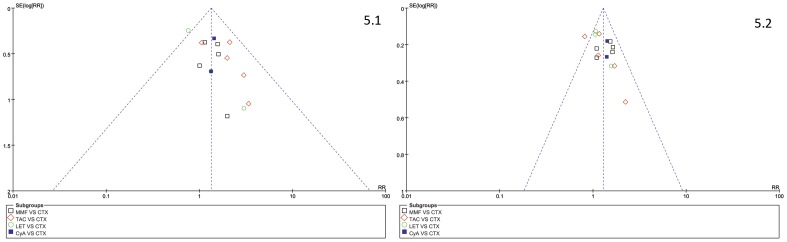
Funnel plot of complete remission (CR; **figure 5**.1) and complete/partial remission (CR/PR; **figure 5**.2) in four comparisons.

The funnel plots (Figure 5) did not show significant visual asymmetry.

We conducted a sensitivity analysis focus on the quality and patients of trials to assess the robustness of this meta-analytical results.

An analysis was performed by excluding low quality trials. As shown in Table 1, the quality score of all included trials is not high, only in one study [11] blindness was used. So we excluded the trials scoring less than 2 points. 2 studies [12], [19] were excluded. The inferior position of cyclophosphamide have not changed in the analyses of “calcineurin inhibitors versus alkylating agents”, on complete remission (CR, RR 1.53, 95% CI 1.06 to 2.20), on complete/partial remission (CR/PR, RR 1.25, 95% CI 1.05 to 1.48). In the analyses of “TAC versus CTX”, the comparison on CR maintained (RR 1.61, 95% CI 1.01 to 2.56), on CR/PR became not statistical significant (RR 1.16, 95% CI 0.95 to 1.41). This sensitivity analysis did not substantially change the results of other comparisons.

## Discussion

Idiopathic membranous nephropathy (IMN) is the most common form of nephrotic syndrome in adults. Immunosuppressive agents acts predominate in its treatment for its benign or indolent course. As single-use glucocorticoids showed no benefit on IMN [3], several immunosuppressive agents in combination with glucocorticoids widely be used in China, namely CTX, CyA, LET, MMF and TAC. There was no good evidence for the choices of immunosuppressive agents in treating nephrotic IMN.

The object of this meta-analysis was to compare the efficacy and safety of different immunosuppressive in the treatment of Chinese adults with nephrotic IMN, providing some updated references to nephrologists for making optimal therapy. By limiting trials conducted in Chinese adults, we aimed to exclude the interference of ethnic differences on the response to immunosuppressive treatment, as some studies [26]–[28] showed that Asian might have better prognosis in IMN compared to Caucasian.

None of the studies involved reported the long-term outcome, like mortality or ESRD requiring initiation of dialysis or kidney transplantation. This analysis only viewed the short-term parameters to evaluate efficacy, including the final proteinuria/serum creatinine/serum albumin values and the therapeutic remission of participants (complete remission, partial remission). Serum creatinine is a value determined by multifactor, and has not showed obvious change during short-term follow up. Final proteinuria and serum albumin has correlation with the therapeutic remission, so the authors mainly analysed the latter. The most frequent definition usually adopted for “partial remission” was proteinuria between 0.3–2.0 g/24 h or decreased to lower by half. For “complete remission” the usual definition was proteinuria of less than 0.3 g/24 h and serum albumin more than 35 g/L and a normal renal function. However these definitions can be heterogeneous.

Cyclophosphamide as a classical immunosuppressive agent used in Chinese nephrotic IMN patients, was compared with other relatively new immunosuppressive agents, including LET, MMF, TAC and CyA. There were heterogeneous in the usage of cyclophosphamide: in 3 trials [10], [18], [19] received daily oral CTX 100 mg/d for 6 months then reduced half for another 6 months; in the other 12 trials, CTX was given intravenously (1g/month, for single dose or divided into two times). Through the comparison “calcineurin inhibitors versus alkylating agents”, IMN patients showed a better treatment response to calcineurin inhibitors. In the analysis of two different agents, tacrolimus was in optimistic position, showing better response than CTX, statistically significant higher rate on inducing remission than CTX, and with tolerable side effects. When compared to CTX, MMF and CyA induced more response but not significant in inducing complete remission, LET shown no significant difference both on complete remission and complete/partial remission. But only 2 studies involving 116 patients compared CyA with CTX, more high quality RCTs were needed to determine their effects. Only one study was included in the analysis on “TAC versus LET” and “MMF versus modified Ponticelli regimen”, both shown no significant difference.

Sensitivity analysis was performed by excluding low quality trials, did not substantially change the main results. This meta’s result “calcineurin inhibitors inducing more remission than alkylating agents” coincided with the earlier meta [3]. “TAC’s favor position” was supported by data from previous TAC monotherapy effect [29]. The funnel plots did not show obvious publishing bias of mainly comparisons.

Short-term duration (6–24 mouths), only one trial [11] used blindness, not large-sample participants (696 in total), absence comparison between some agents(mostly compared to CTX), no advanced subgroup analyses of different level proteinuria (only definition was “nephrotic”) led to limitations of this meta. The probable explain for non-blindness was those agents have a relative high adverse rate and blood drug concentration level need to be checked. CTX have been compared in most studies, possibly for its classical position.

In conclusion, based on Chinese adults and short duration RCTs, calcineurin inhibitors, especially TAC, showed superior potency to induce remission in nephrotic IMN with tolerable adverse effects, compared to alkylating agent (CTX).

## References

[1] Schieppati A, Mosconi L, Perna A, Mecca G, Bertani T, et al (1993) Prognosis of untreated patients with idiopathic membranous nephropathy. N Engl J Med 329: 85–89. 8510707 10.1056/NEJM199307083290203

[2] Honkanen E, Tornroth T, Gronhagen-Riska C (1992) Natural history, clinical course and morphological evolution of membranous nephropathy. Nephrol Dial Transplant 7 Suppl 135–41. 1337181

[3] Schieppati A, Perna A, Zamora J, Giuliano GA, Braun N, et al.. (2004) Immunosuppressive treatment for idiopathic membranous nephropathy in adults with nephrotic syndrome. Cochrane Database Syst Rev: CD004293. 10.1002/14651858.CD004293.pub2 15495098

[4] Jha V, Ganguli A, Saha TK, Kohli HS, Sud K, et al (2007) A randomized, controlled trial of steroids and cyclophosphamide in adults with nephrotic syndrome caused by idiopathic membranous nephropathy. J Am Soc Nephrol 18: 1899–1904. 17494881 10.1681/ASN.2007020166

[5] Ponticelli C, Altieri P, Scolari F, Passerini P, Roccatello D, et al (1998) A randomized study comparing methylprednisolone plus chlorambucil versus methylprednisolone plus cyclophosphamide in idiopathic membranous nephropathy. J Am Soc Nephrol 9: 444–450. 9513907 10.1681/ASN.V93444

[6] Ponticelli C, Zucchelli P, Passerini P, Cagnoli L, Cesana B, et al (1989) A randomized trial of methylprednisolone and chlorambucil in idiopathic membranous nephropathy. N Engl J Med 320: 8–13. 2642605 10.1056/NEJM198901053200102

[7] Ponticelli C, Zucchelli P, Passerini P, Cesana B, Locatelli F, et al (1995) A 10-year follow-up of a randomized study with methylprednisolone and chlorambucil in membranous nephropathy. Kidney Int 48: 1600–1604. 8544420 10.1038/ki.1995.453

[8] Jadad AR, Moore RA, Carroll D, Jenkinson C, Reynolds DJ, et al (1996) Assessing the quality of reports of randomized clinical trials: is blinding necessary? Control Clin Trials 17: 1–12. 8721797 10.1016/0197-2456(95)00134-4

[9] Chan TM, Lin AW, Tang SCW, Qian JQ, Lam MF, et al (2007) Prospective controlled study on mycophenolate mofetil and prednisolone in the treatment of membranous nephropathy with nephrotic syndrome. Nephrology 12: 576–581. 17995584 10.1111/j.1440-1797.2007.00822.x

[10] Chen M, Li H, Li XY, Lu FM, Ni ZH, et al (2010) Tacrolimus combined with corticosteroids in treatment of nephrotic idiopathic membranous nephropathy: A multicenter randomized controlled trial. American Journal of the Medical Sciences 339: 233–238. 20220333 10.1097/MAJ.0b013e3181ca3a7d

[11] Xu J, Zhang W, Xu Y, Chen N (2010) A double-blinded prospective randomised study on the efficacy of corticosteroid plus cyclophosphamide or FK506 in idiopathic membranous nephropathy patients with nephrotic syndrome. Nephrology 15: 43.20586947

[12] Bai GZ, Fan W, Yuan YJ, Wang JH, Zhang SH (2011) An observation of tacrolimus in combination with low dose steriod in idiopathic membranous nephropathy. Journal of Guiyang College of Traditional Chinese Medicine 33: 52–53.

[13] Chen WZ, Chen DJ, Xu GB (2009) An observation of tacrolimus in idiopathic membranous nephropathy treatment The Journal of Practical Medicine. 25: 1674–1675.

[14] Li GF, Liu T, Bao BY (2011) Comparison on the therapeutic effect of cyclosporin A and cyclophosphamide in the creatment of idiopathic membranous nephropathy. Chinese Journal of Integrated Traditional and Western Nephrology 12: 522–525.

[15] Liu J, Li D (2009) The study on the treatment of idiopathic membranous nephropathy with tacrolimus. graduation dissertation.

[16] Wu QX, Gong ZF (2011) Middle or small dose tacrolimus in the treatment of 20 membranous nephropathy patients. China Pharmacist 14: 115–117.

[17] An WW, Tu YK, Wang T (2009) Clinical research of mycophenolate mofetil and glucocorticoid in treatment of membranous nephropathy. Occupation and Health 25: 2009–2010.

[18] Li MX, Song JW, Yu YW, Shi XY (2004) Effects of mycophenolate mofetil in combination of prednesone on the early stage of membranous nephropathy. journal of Clinical nephrology 4: 160–162.

[19] Ren Y, Hu ZX, Luo YH, Shan W (2011) Clinical observation of mycophenolate mofetil combined with low-dose prednisone on idiopathic membranous nephropathy. Journal of modern Chinese doctor 49: 73–74.

[20] Zhang W, Zhang XT, Ma JW, Liu HF (2011) Mycophenolate mofetil plus small dose steroid in the treatment of membranous nephropathy. Chinese Journal of Integrated Traditional and Western Nephrology 12: 59–60.

[21] Zhou W, Zhang WX, Zhang ZM, Zhang ZQ, Shi XY (2009) Mycophenolate mofetil in the treatment of 20 membranous nephropathy patients. Journal of Clinical Internal Medicine 26: 479–481.

[22] Zhou W, Zhang WX, Zhang ZM, Zhang ZQ, Shi XY (2009) Clinical trial on the effect of leflunomide in treating primary membranous nephropathy. Sichuan Medical Journal 30: 1889–1891.

[23] Zhu KY, Bi CY (2009) The clinical efficacy observation of leflunomide in the treatment of membranous nephropathy. China Practical Medicine 4: 99–100.

[24] Li GF, Liu T, Bao BY (2011) Efficacy comparison of lefluomide and cyclophosphamine treatment on idiopathic nephropathy. Chinese Journal of Integrated Traditional and Western Nephrology 12: 872–874.

[25] Sun GD, Xu ZG, Luo P, Miao LN (2008) The clinical efficacy of tacrolimus in the treatment of idiopathic membranous nephropathy. Chinese Journal of Gerontology 28: 469–471.

[26] Shiiki H, Saito T, Nishitani Y, Mitarai T, Yorioka N, et al (2004) Prognosis and risk factors for idiopathic membranous nephropathy with nephrotic syndrome in Japan. Kidney Int 65: 1400–1407. 15086481 10.1111/j.1523-1755.2004.00518.x

[27] Donadio JV Jr, Torres VE, Velosa JA, Wagoner RD, Holley KE, et al (1988) Idiopathic membranous nephropathy: the natural history of untreated patients. Kidney Int 33: 708–715. 3367560 10.1038/ki.1988.56

[28] Reichert LJ, Koene RA, Wetzels JF (1998) Prognostic factors in idiopathic membranous nephropathy. Am J Kidney Dis 31: 1–11. 9428445 10.1053/ajkd.1998.v31.pm9428445

[29] Praga M, Barrio V, Juarez GF, Luno J (2007) Tacrolimus monotherapy in membranous nephropathy: a randomized controlled trial. Kidney Int 71: 924–930. 17377504 10.1038/sj.ki.5002215

